# Colony Level Prevalence and Intensity of *Nosema ceranae* in Honey Bees (*Apis mellifera* L.)

**DOI:** 10.1371/journal.pone.0163522

**Published:** 2016-09-22

**Authors:** Cameron J. Jack, Hannah M. Lucas, Thomas C. Webster, Ramesh R. Sagili

**Affiliations:** 1 Department of Horticulture, Oregon State University, Corvallis, Oregon, United States of America; 2 Department of Entomology and Nematology, University of Florida, Gainesville, Florida, United States of America; 3 College of Agriculture, Food Science & Sustainable Systems, Kentucky State University, Frankfort, Kentucky, United States of America; University of California San Diego, UNITED STATES

## Abstract

*Nosema ceranae* is a widely prevalent microsporidian parasite in the western honey bee. There is considerable uncertainty regarding infection dynamics of this important pathogen in honey bee colonies. Understanding the infection dynamics at the colony level may aid in development of a reliable sampling protocol for *N*. *ceranae* diagnosis, and provide insights into efficient treatment strategies. The primary objective of this study was to characterize the prevalence (proportion of the sampled bees found infected) and intensity (number of spores per bee) of *N*. *ceranae* infection in bees from various age cohorts in a colony. We examined *N*. *ceranae* infection in both overwintered colonies that were naturally infected with *N*. *ceranae* and in quadruple cohort nucleus colonies that were established and artificially inoculated with *N*. *ceranae*. We also examined and quantified effects of *N*. *ceranae* infection on hypopharyngeal gland protein content and gut pH. There was no correlation between the prevalence and intensity of *N*. *ceranae* infection in composite samples (pooled bee samples used for analysis). Our results indicated that the prevalence and intensity of *N*. *ceranae* infection is significantly influenced by honey bee age. The *N*. *ceranae* infection prevalence values from composite samples of background bees (unmarked bees collected from four different locations in a colony) were not significantly different from those pertaining to marked-bee age cohorts specific to each sampling date. The foraging-aged bees had a higher prevalence of *N*. *ceranae* infection when compared to nurse-aged bees. *N*. *ceranae* did not have a significant effect on hypopharyngeal gland protein content. Further, there was no significant difference in mean gut pH of *N*. *ceranae* infected bees and non-infected bees. This study provides comprehensive insights into *N*. *ceranae* infection dynamics at the colony level, and also demonstrates the effects of *N*. *ceranae* infection on hypopharyngeal gland protein content and midgut pH.

## Introduction

The European honey bee (*Apis mellifera*), the world’s most economically important and intensely managed pollinator is currently facing many stressors. Parasites and pathogens are among those stressors. Honey bee colonies are often suggested as the definitive example of a superorganism [1], a complicated biological system composed of many individuals that function as one whole. As such, the pathology of disease at the colony level may exhibit different characteristics than that of the same disease in an individual bee. The distribution of the microsporidian gut parasite *Nosema ceranae* within a colony may well reflect this dichotomy. *Nosema ceranae* infection of honey bees has been shown to increase colony loss [2, 3] and there is evidence suggesting it to be a contributing factor in collapsing colonies [4–6]. Therefore, a better understanding of *N*. *ceranae* infection dynamics at the whole colony level may ultimately lead to more successful management practices and fewer colony losses.

*Nosema ceranae* was first described in the Asian honey bee, *Apis cerana*, in 1996 [7], and first found infecting *A*. *mellifera* in 2006 [8–10]. It is thought that this microsporidian began parasitizing *Apis mellifera* relatively recently in comparison to *A*. *cerana*, but since its first discovery in the European honey bee, *N*. *ceranae* presence has been documented in *A*. *mellifera* samples collected as far back as 1985 [11–13].

Once ingested, *N*. *ceranae* spores germinate inside the host’s midgut and inject their sporoplasm in to epithelial cells lining the gut [7, 14, 15]. Intracellular proliferation of *N*. *ceranae* will eventually burst the epithelial cell membrane, thus destroying the cells [16]. The combined effects of cell destruction and robbing of energy by parasites often manifest increased hunger levels [17] and diminished nestmate food sharing via trophallaxis [18] in infected honey bees. *Nosema ceranae* infection has also been shown to alter the expression of portions of the midgut proteome [19], promote precocious foraging [20], modify vitellogenin titres and queen mandibular pheromones in queens [21], decrease immune functions [22] and reduce longevity [23].

Another area of concern regarding *N*. *ceranae* infections is the potentially poor development of the hypopharyngeal glands in infected honey bees. In nurse bees, hypopharyngeal glands produce brood food, a protein-rich diet supplied to honey bee larvae [24]. Hypopharyngeal glands also synthesize enzymes involved in the conversion of sucrose to simple sugars and honey [25, 26]. Therefore, any detrimental impact on the development of the hypopharyngeal glands could significantly affect brood rearing and ultimately the colony growth. It is known that the presence of *Nosema apis* decreases hypopharyngeal gland protein content [27, 28] and alters the composition of secretions [29]. *Nosema ceranae* specific PCR signals were detected in the hypopharyngeal gland tissue [30] and Alaux et al. [31] demonstrated that *Nosema* infection induces gland atrophy. Recent research has also shown that *N*. *ceranae* parasitism results in decreased protein content in hypopharyngeal glands [32]. However, currently it is not well understood if and how different levels of *N*. *ceranae* infection influence hypopharyngeal gland protein production.

Gut pH is a critical factor in insect health; it is essential for digestion, as well as maintaining optimal gut microflora. But little is known about the relationship between gut pH and *Nosema* infection, as only a few preliminary studies, published decades ago, describe their interactions. Early literature from Bailey [33] suggests that gut pH alone does not trigger *Nosema apis* germination, while Jaronski [34] concluded that *Nosema algerae* spore germination was influenced by pH stimuli. Additionally, the effect of pH on priming *Nosema michaelis* spores for germination is described by Weidner [35]. Ishihara [36] also described a higher rate of polar filament extrusion, an important step in spore reproduction, in the microsporidium *Glugea fumiferanae*, at higher pH. Hence it appears that it may be possible for a specific gut pH level to provide an environment conducive for spore germination. A better understanding of how honey bee gut conditions influence proliferation of *N*. *ceranae* would greatly advance our efforts in mitigating *N*. *ceranae* infections.

Some studies have shown that the intensity of *N*. *ceranae* infection varies greatly among bees in an infected colony—most bees apparently exhibiting no signs of infection, while others show extremely high spore intensities [37, 38, 39]. Therefore, a few highly infected bees in a sample may greatly misrepresent the level of infection, possibly providing the false perception of poor colony health. Conversely, without understanding how *Nosema* infection is distributed throughout the colony, there is the potential to inadvertently collect a sample comprised mainly of bees from an age cohort with far lower infection levels than most other bees in the colony. Once again, such a sample would produce an inaccurate assessment of *N*. *ceranae* infection level; this time vastly underestimating the infection. Currently, there is no reliable method of sampling honey bee colonies for *N*. *ceranae* infection. Many studies have used the number of spores from composite samples—samples of bees pooled together and analyzed as one big sample—to determine colony infection status [40–43]; though others have suggested that measuring the proportion of infected individuals may be more accurate [44, 45].

Forager bees are likely to have higher *Nosema* infection levels than nurse bees [2, 38, 44–47]; hence many studies have diagnosed *Nosema* infections from samples comprised solely of foragers taken from the hive entrance. However, other studies involving *Nosema* sampling have demonstrated that bees obtained from the brood area also exhibit significant *Nosema* infection [13, unpublished data Sagili et al.]. There is an urgent need to thoroughly understand the infection dynamics at the colony level, which may, in turn, aid in developing a reliable sampling protocol for *N*. *ceranae* diagnosis, and may also provide useful insights into efficient treatment strategies for *Nosema* disease.

Here we sought to determine the infection dynamics (prevalence and intensity) of *N*. *ceranae* at the colony level. To achieve this objective we set up two separate rigorous experiments. In the first experiment, we examined the *N*. *ceranae* infection in large, overwintered colonies that were naturally infected with *N*. *ceranae*. In the second experiment, we observed the prevalence and intensity of *N*. *ceranae* in quadruple cohort nucleus colonies that, once established, were artificially inoculated with *N*. *ceranae*. The primary objective of both experiments was to characterize the prevalence (proportion of the sampled bees found to be infected) and intensity (number of spores per bee) of *N*. *ceranae* infection in infected bees from various age cohorts in a colony. We also examined and quantified potential effects of *N*. *ceranae* infection on hypopharyngeal gland protein content and gut pH.

## Materials and Methods

No formal permits were required for field collections or laboratory analysis because honey bee research is not regulated by animal use committees such as the Institutional Animal Care and Use Committee (IACUC).

### Experiment 1

Five colonies naturally infected with *N*. *ceranae* were selected in July 2013 from a single Oregon State University apiary (Corvallis, OR, USA). Prior to the experiment, we identified the *Nosema* species in all five colonies as *N*. *ceranae* using the DNA analysis methods of Hamiduzzaman et al. [48]. Two to three capped combs with emerging bees were collected from each of the five colonies and incubated in the laboratory under simulated hive conditions (33°C, 55% RH) for bee emergence. Incubating brood combs were checked for emerging bees 16 hours later. Newly emerged bees were color-coded by colony with a dot of Testors^TM^ enamel paint on the thorax. Once at least 500 newly emerged bees per colony were painted, they were returned to their original colonies. Fifty recently emerged bees per colony were also retained to establish a baseline infection level of *N*. *ceranae*.

From each of these five experimental colonies, fifty marked (painted) bees were collected during midday 8‒11 days post-emergence and again at 22‒25 days post-emergence. These sampling periods represent the nursing and foraging phases, respectively, of a worker bee, as honey bees exhibit temporal polyethism. Each time when collecting these fifty marked bees, an additional sample of fifty unmarked bees (background bees) was also collected from each colony. Hence, we had three different sampling events pertaining to background bees to examine temporal differences. Smart and Sheppard [38], in their 2012 study from which our methods were adapted, collected such background bee samples from the inner cover only. However, our samples of background bees consisted of ten individuals taken from the brood area, ten from the cover (lid), ten from the colony entrance and ten each from outer combs. The infection intensity and prevalence of *Nosema ceranae* in these random samples of mixed aged bees were compared to the infections of the marked bees of known age.

All collected marked bees were analyzed individually. The prevalence and intensity of *Nosema* infection in all collected bees were determined following the light microscopy techniques described by Cantwell [49]. Briefly, each bee abdomen was macerated by mortar and pestle in 1 ml of distilled water. A 10 μl drop of that solution was placed on a hemocytometer (Cat # 3200, Hausser Scientific, PA, USA), from which spores were counted at 400X magnification. Although background bees were collected as composite samples, they too were analyzed individually. This permitted us to observe the prevalence and intensity of infection in the bees comprising a composite sample, which is usually not known because all bees are typically analyzed as one pooled sample. Furthermore, individually analyzing bees from the composite samples allowed us to observe if any significant differences exist between the results derived from sampling bees of known ages and traditional composite samples.

### Experiment 2

Eight nucleus colonies were established for this experiment. Prior to establishing these experimental nucleus colonies, we collected bee samples from several colonies at Oregon State University’s apiaries (Corvallis, OR, USA) in July 2013 and May 2014, and examined them for the presence of *Nosema* using light microscopy as described above. Then, during each year (2013 and 2014), four colonies that were determined to be *Nosema*-free were randomly selected to serve as foster colonies for the bees that were used to establish the experimental nucleus colonies. The foster colonies were two story standard Langstroth hives with approximately 50,000 bees that were used for releasing the marked bees for aging and to be collected later when needed (as described in the next paragraph). The nucleus colonies were established according to standard methods used for triple-cohort colonies [50–52]; however, we created an additional fourth cohort. Here onwards, we refer to these experimental nucleus colonies as quadruple-cohort colonies. Each of the eight quadruple-cohort colonies (eight replications) was composed of 1,000 bees from each of the following age groups: (1) one day old bees (2) one week old bees (3) two week old bees (4) three week old bees. The detailed procedure for establishing these four age groups is furnished in the next paragraph. For the purposes of brevity, cohorts will herein be referred to by their age at the time of colony establishment (as described above) rather than their actual age when they were sampled for analysis. Because bees were sacrificed for analysis two weeks after inoculation (see below), each cohort was two weeks older at the time of analysis than at the time of colony establishment (Table 1).

**Table 1:** Summary of the age cohorts at time of quadruple-cohort colony establishment and analysis two weeks later.

Age at colony establishment	Age at time of analysis
1 Day	2 Weeks
1 Week	3 Weeks
2 Weeks	4 Weeks
3 Weeks	5 Weeks

For establishing nucleus colonies, capped combs with emerging bees were obtained from honey bee colonies headed by sister queens and were incubated in the lab under simulated hive conditions (33°C, 55% RH) for bee emergence. Sister queen colonies were used to control any variation in *Nosema* infection attributed to genetics of the bees. Incubating brood combs were checked after 16 hours for emerging bees. Beginning three weeks prior to establishing our quadruple-cohort colonies (Week ‒3), newly emerged bees were painted on the thorax with Testors™ enamel paint to indicate the appropriate age cohort and were then released into their designated *Nosema*-free foster colonies for aging. This same procedure was performed at two weeks prior (Week ‒2) and one week prior (Week ‒1) to establishing the nucleus colonies. Each cohort of bees was painted a unique color. In a given year, at least 2,000 newly emerged bees per target cohort were released into each of four foster colonies to ensure that 1,000 painted bees per cohort could be easily collected and added to each corresponding nucleus colony at time of establishment (Day 0).

Three weeks after the first cohort of bees were placed in the foster colonies, 1,000 painted bees from each cohort were vacuumed out of a given foster colony with a BioQuip Insect Vac™ (Cat # 2820GA, BioQuip Products Inc., CA, USA) and placed inside a nucleus colony with closed entrance. Later, on the same day, 1,000 newly emerged painted bees were also placed into each of these nucleus colonies. Each quadruple-cohort colony contained three empty combs, one honey comb and one comb containing uncapped brood. All the combs used in establishing the nucleus colonies were obtained from colonies that were determined to be free from *N*. *ceranae* infection.

The day after establishing nucleus colonies (Day 1) entrance blockers on each of the nucleus colonies were removed so that the bees would forage freely. Also, on Day 1, any existing non-painted bees that were accidentally vacuumed up along with painted bees were removed, and a queen in a sealed queen cage was placed inside each colony. On Day 2, the sealant on the queen cage was removed, so that bees could release the queen on their own. On Day 3, the *N*. *ceranae* spores were fed to bees in all the nucleus colonies through mass inoculation methods as described below. Two weeks after inoculation, colony entrances for all the experimental nucleus colonies were blocked late in the evening and the colonies were placed inside a −20°C freezer for two days. The sacrificed bees were then removed from frozen combs, separated into respective cohorts based on respective paint color, and counted. A summary of the above procedures, used to establish the infected, quadruple-cohort colonies, is provided in Table 2.

**Table 2:** Summary of the methods used to establish all experimental quadruple-cohort colonies in 2013 and 2014.

Time relative to nucleus colony establishment	Procedure
Week −3	Bees emerging from selected incubated combs were painted green to identify them as 3 weeks old and then placed in foster colonies.
Week −2	Bees emerging from incubated combs were painted blue to identify them as 2 weeks old and then placed in foster colonies.
Week −1	Bees emerging from incubated combs were painted white to identify them as 1 week old and then placed in foster colonies.
**Day 0**	Experimental nucleus colonies were made:
	• Painted bees moved from foster colonies to nucleus colonies.
	• Newly emerged bees were painted red to identify them as 1 day old and moved straight from incubated comb to nucleus colonies.
Day 1	• Entrance blockers and unpainted bees were removed from nucleus colonies.
	• Caged queens were added to nucleus colonies.
Day 2	Plugs of queen cages were removed.
Day 3	Nucleus colonies were inoculated with *Nosema ceranae* via sugar syrup.
Day 17	All bees in all nucleus colonies were frozen for future analyses.

Prior to inoculation, DNA analysis was performed using the methods of Hamiduzzaman et al. [48] to confirm that only *N*. *ceranae* spores were used in the inoculum. The spore concentration of the inoculating solution was formulated following the methods of Fries et al. [53]. Spores were purified through centrifugation and the *N*. *ceranae* solution was prepared in 100 ml of 50% (v/v) sugar syrup at a concentration sufficient to inoculate 4,000 bees with an average of 10,000 spores each. We provided the spore inoculant to experimental colonies via inverted mason jars placed over a hole in the nucleus colony lid. After the spore inoculant was consumed completely, bees were fed 300 ml of *Nosema*-free sugar syrup ad libitum.

The prevalence and intensity of *Nosema* infection in each bee were determined by light microscopy techniques of Cantwell [49] described previously. Similarly, the queens from each of the eight nucleus colonies were also tested for *Nosema* infection. The resulting macerated gut extracts were stored at −20°C for further pH analysis and *Nosema* species confirmation.

#### Confirmation of *Nosema ceranae* via PCR

Gut samples used to determine the prevalence and intensity of *Nosema* infection were saved at −20°C if spores were detected. In order to confirm the species of *Nosema* spores found in experimental bees, we randomly selected the *Nosema*-positive gut samples from one of the four age cohorts from every colony, such that each age cohort was selected once within each year’s set of colonies. From these randomly selected *Nosema*-positive gut samples, we extracted DNA samples using phenol:chloroform extraction and alcohol precipitation. Briefly, 2 ml of the bee gut-spore homogenate were well mixed with an equal volume of phenol:chloroform:isoamyl alcohol (25:24:1), further macerated by mortar and pestle, and centrifuged at 12,000 rpm for 3 minutes, at room temperature. The resulting upper aqueous layer was then vigorously mixed with an equal volume of phenol:chloroform:isoamyl alcohol solution and centrifuged again. The new upper aqueous layer was gently mixed with an equal volume of room temperature 100% isopropyl alcohol, incubated at room temperature for 15 minutes and centrifuged as before. Finally, the resulting pellet was washed with 1 ml of ice cold 80% ethanol, centrifuged again as before, air dried for 1 hour at room temperature, and resolubilized in 20 μl of chilled, autoclaved distilled water. The nucleic acid concentration of each DNA sample was quantified on a Take3 micro-volume plate in a Biotek Synergy 2 plate reader (BioTek Instruments, Inc., VT, USA) and then stored at −20°C until time of PCR reactions.

DNA samples were used in the co-amplification of the 16S rRNA gene of *Nosema apis* and *Nosema ceranae* (NAPIS and MITOC, respectively) and the honey bee ribosomal protein S5 gene (RpS5) in the same multiplex PCR reaction. Each 50 μl reaction contained 5 μl of 10X Ex Taq® Buffer (Cat # RR001A, Takara Bio Inc., Shiga, Japan), 4 μl of 2.5 mM dNTPs (Cat # RR001A, Takara Bio Inc.), 1 μl of 10 μM of each primer (MITOC and RpS5: BioNeer Corp., CA, USA; NAPIS: IDT Inc., IA, USA), 0.25 μl of 5U/ μl Ex Taq® DNA Polymerase (Cat # RR001A, Takara Bio Inc.), approximately 100 ng of template DNA and sterile distilled water to bring to volume. All PCR reactions were performed in a GeneAmp® PCR System 9700 thermocycler (Applied Biosystems, CA, USA), programmed as follows: 94°C for 2.5 min, followed by 10 cycles of 94°C for 15 sec, 62°C for 30 sec, and 72°C for 45 sec; then 20 cycles of 94°C for 15 sec, 62°C for 30 sec, and 72°C for 50 sec; with a final extension step of 72°C for 7 min (modified from Martin-Hernandez et al. [54] and Hamiduzzaman et al. [48]).

Primers amplifying a 218 bp product within the *Nosema ceranae* 16S rRNA gene were: MITOC-F (5’ CGGCGACGATGTGATATGAAAATATTAA 3’) and MITOC-R (5’ CCCGGTCATTCTCAAACAAAAAACCG 3’) [53]. Primers amplifying a 788 bp product within the *Nosema apis* 16S rRNA gene were: NAPIS-F (5’ GCATGTCTTTGACGTACTATG 3’) and NAPIS-R (5’ CTCAGATCATATCCTCGCAG 3’). The NAPIS primers were designed based on ClustalW (http://www.ebi.ac.uk/Tools/msa/clustalw2/) alignments of *Nosema* species sequences published in the GenBank database (http://www.ncbi.nlm.nih.gov/GenBank/): *Nosema apis* accession numbers DQ235446, U76706, U97150, U26534, X73894, X74112; *Nosema ceranae* accession numbers DQ329034, U26533, DQ078785, DQ286728. As a standard control, a 115 bp segment of the *Apis mellifera* house-keeping gene RpS5 was amplified in all samples using the primers RpS5-F (5’ AATTATTTGGTCGCTGGAATTC 3’) and RpS5-R (5’ TAACGTCCAGCAGAATGTGGTA 3’) [55]. Reference DNA samples for *Nosema apis* and *Nosema ceranae* were obtained from the Solter lab at University of Illinois Urbana-Champaign. The PCR products were separated by electrophoresis in a 2% agarose gel stained with 5% Midori Green (Cat # MG03, Nippon Genetics, Düren, Germany) and run at 100 volts. For reference, a 100 bp DNA ladder (Cat # G2101, Promega Corp., WI, USA) was included.

#### Hypopharyngeal gland protein quantification

The heads of all bees analyzed for *Nosema* (as described previously) were placed in individual 0.5-ml microcentrifuge tubes and stored at −20°C until time of hypopharyngeal gland protein quantification. Only bees from the four 2013 quadruple-cohort colonies were used for hypopharyngeal gland protein analysis. Sixty heads from each age cohort (from all four colonies combined) were separated into three groups of 20 based on *N*. *ceranae* infection levels (*Nosema* free, low infection, high infection). So, overall 240 (60 bee heads x 4 age cohorts) pairs of hypopharyngeal glands were used for protein quantification. Infection levels were based on the availability of bees within a similar range of spore intensity as follows: *Nosema* free bees with absolutely no spores detected; bees with low level of infection (between 7.5 x 10^5^ and 1.2 x 10^6^ spores/bee); bees with high infection level (between 3.685 x 10^7^ and 5.76 x 10^7^ spores/bee).

We collected the complete pair of hypopharyngeal glands from each of these stored heads. Each honey bee head was placed on a petri dish with wax surface and submerged in few drops of distilled deionized water. Next, using a stereomicroscope the frons were gently removed to expose the hypopharyngeal glands below. The hypopharyngeal glands were then carefully removed from each individual bee head with a fine tip forceps and were stored separately in 1.5-ml microcentrifuge tubes containing 5 μl PBS (Phosphate Buffered Saline, Sigma-Aldrich) and stored at −80°C until time of analysis. During protein analysis we added 115μl of PBS to each pair of glands (for a total PBS volume of 120 μl) and homogenized them with one 3-mm tungsten carbide bead in a Qiagen® TissueLyser II (20 oscillations/sec., 2 x 45 sec.). The samples were then centrifuged at room temperature for 2 minutes at 13,300 rpm to pellet the debris.

We quantified the protein content of the resulting supernatant from each homogenized sample with the Pierce™ Biotech BCA Assay Kit (Cat # 23225, Thermo-Scientific, IL, USA) microplate procedure. These samples were homogenized in PBS; therefore, PBS was used as the blank and the diluent for the Bovine Serum Albumin (BSA) standards. BSA standard solutions were stored at −20°C and used for several consecutive days. We loaded 25 μl of each sample, standard and blank into each of two duplicate wells on a 96-well plate. Plating this volume allows the assay to have a protein detection range of 25‒2000 μg/ml. After addition of the BCA working reagent (200 μl/well), the plate was shaken for 30 seconds on a microplate spectrophotometer (BioTek Synergy 2, Gen5 2.00 software). The plate was then incubated, uncovered, for 30 minutes at 37°C, followed by 12 minutes, covered, at 4°C. Absorbance at 562 nm was measured using the same spectrophotometer as above and protein content in μg/bee was calculated from the resulting standard curve. Due to a slight dilution error in the samples on one plate (150 μl of PBS were added to each pair of glands), the resulting μg/ml outputs for samples of this plate were adjusted to compensate for the greater dilution, thus allowing them to be analyzed and reported on the same scale as all other samples.

#### Gut pH Analysis

For each of the eight experimental colonies, 50 random *Nosema* positive and 50 random *Nosema* negative macerated gut samples from 3 week, 2 week and 1 week old cohorts were preserved for pH measurement. Samples from the one day old cohort were not retained for pH analysis due to the low prevalence of *Nosema* infection. For a given nucleus colony and cohort, each of the 50 random *Nosema* positive and *Nosema* negative gut samples mentioned above were collected in a set of five vials (ten 1-ml macerated samples per vial), for a total of 30 vials per colony (3 cohorts x 2 samples x 5 vials). Samples were stored at −20°C until time of analysis, when they were thawed and the pH in each vial was measured using a Waterproof pH EcoTester™ (Cat # WD-35423-10, Oakton Instruments, IL, USA).

### Statistical Analyses

#### Experiment 1

All statistical analyses were performed using the statistical software SAS 9.3. *N*. *ceranae* prevalence and intensity data were analyzed using general linear models (PROC GLM; SAS 9.3) for differences among the sampling periods. Intensity data were log transformed to meet normality assumptions. Prevalence data was normally distributed, hence was not transformed. Mean separations were performed using least significant difference (LSD) test (P < 0.05). After statistical analysis, data were back-transformed as needed for presentation herein.

#### Experiment 2

All statistical analyses were performed using the statistical software SAS 9.3. *N*. *ceranae* prevalence and intensity data were analyzed using generalized linear models (PROC GLIMMIX; SAS 9.3) for differences among age cohorts. Both prevalence and intensity data were log transformed to meet normality assumptions. Mean separations were performed using least significant difference (LSD) test (P < 0.05). Hypopharyngeal gland protein content data were analyzed using mixed linear models with age and infection level as fixed effects and different honey bee colonies as random effects (PROC MIXED; SAS 9.3). Mean separations were again performed using least significant difference (LSD) test (P < 0.05). Hypopharyngeal gland protein content data were log transformed to meet normality assumptions. After statistical analysis, data were back-transformed as needed for presentation herein. The gut pH data were compared using an independent samples t-test.

## Results

### Experiment 1

#### *Nosema ceranae* prevalence and intensity

**Fig 1:**
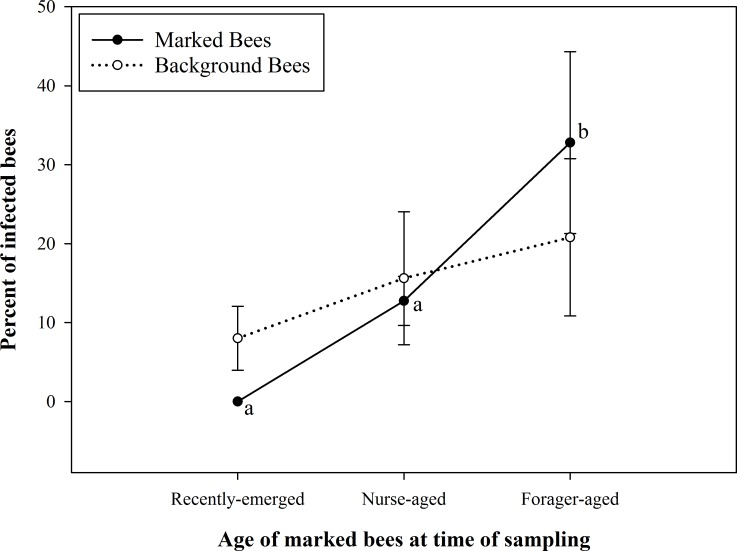
Prevalence of *Nosema ceranae* infection in samples of known-age bees (marked) and mixed age background bees. Shown are mean ± SE *Nosema ceranae* infection levels of marked bees and background bees at each age group/sampling event. Marked bees were recently emerged bees at the first sampling event, nurse-aged bees at the second sampling event and forager-aged bees when the third sample was taken. Mean infection prevalence between marked (known-age) and background (unknown, mixed-age) bees on corresponding collection days were not significantly different (P > 0.05). Mean prevalence of infection was significantly higher in marked bees sampled on date 3 than on dates 1 and 2, indicated by different letters (P = 0.0043).

The results of *N*. *ceranae* prevalence (proportion of infected bees) are summarized in Fig 1. The prevalence of *N*. *ceranae* infection was significantly different between some age cohorts (*F*_5, 24_ = 4.92; *P* = 0.0043). *N*. *ceranae* infection was not detected in any of the recently emerged bees (Table 3). Foraging-aged bees that were marked had a significantly higher prevalence of *N*. *ceranae* infection than both nurse-aged bees (marked) and recently emerged bees (marked). However, there was no significant difference in levels of *Nosema* prevalence between nurse-aged bees and recently emerged bees that were marked. The prevalence of *N*. *ceranae* infection in background bees was not significantly different between sampling dates; nor did it differ from that of corresponding marked bees on any of the sampling dates. Lastly, there was no significant correlation (r = 0.29, *P* > 0.05) between the percentage of *N*. *ceranae* infected bees (prevalence) and infection level (intensity) in a composite sample (multiple bees of unknown age collected from different locations in a colony).

**Table 3:** Summary of *Nosema ceranae* infection levels and percent of individual bees infected with *N*. *ceranae* in each of the five experimental colonies.

Colony #	Age Group	n sampled	n infected	% infected	NSI (x10^6^)	NSS (x10^6^)
1	RE	50	0	0	0	0
	Nurse	26	2	7.7	0.93	0.07
	Forager	50	3	6	41.77	2.56
	BG	150	7	4.7	19.19	0.9
2	RE	50	0	0	0	0
	Nurse	50	9	18	0.35	0.06
	Forager	50	15	30	2.01	0.6
	BG	150	5	3.3	5.54	0.18
3	RE	50	0	0	0	0
	Nurse	50	3	6	6.53	0.39
	Forager	50	9	18	28.32	5.1
	BG	150	12	8	14.25	1.14
4	RE	50	0	0	0	0
	Nurse	50	5	10	0.11	0.01
	Forager	50	18	36	7.08	2.55
	BG	150	24	16	9.27	1.48
5	RE	50	0	0	0	0
	Nurse	50	11	22	1.33	0.29
	Forager	50	37	74	1.52	1.13
	BG	150	61	40.7	2.89	1.18

RE = recently emerged bees, BG = background bees collected from the inner hive cover, outer frames, hive entrance and brood area at the same time as each group (RE, Nurse-aged bees, Foraging-aged bees), NSI = mean number of spores across infected bees, NSS = mean number of spores across all sampled bees (composite sample).

**Fig 2:**
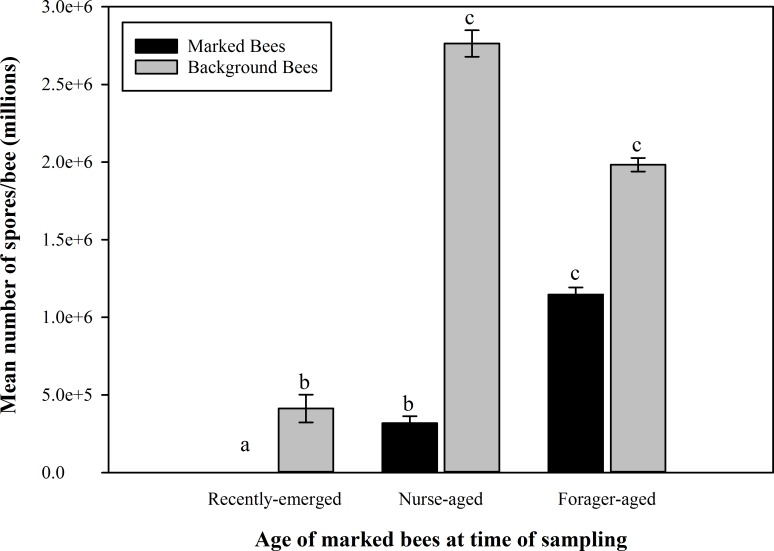
*Nosema ceranae* infection intensity in samples of known-age bees (marked) and mixed-age background bees. Shown are mean ± SE *Nosema ceranae* infection intensities of marked bees and background (unknown, mixed-age) bees at each age group/sampling event. Marked bees were recently emerged bees at the first sampling event, nurse-aged bees at the second sampling event and forager-aged bees at the third sampling event. There were significant differences among the age cohorts, as indicated by different letters (P < 0.01).

The mean *N*. *ceranae* infection intensities were significantly different between all age cohorts (*F*_5, 221_ = 243.64; *P* < 0.01). Foraging-aged bees had significantly higher *N*. *ceranae* spore intensities when compared to both nurse-aged bees and recently emerged bees. Nurse-aged bees had significantly higher spore intensities than recently emerged bees. The results of *N*. *ceranae* intensity (number of spores per infected bee) are summarized in Fig 2. The mean infection intensities in background bees were significantly different between some sampling dates. Background bees from the second and third sampling dates, while not significantly different from each other, demonstrated higher intensities than bees from the first sampling date. Moreover, the background bees from the first and second sampling dates had higher mean intensities than the marked bees sampled at the same time—recently emerged bees and nurse-aged bees, respectively. In background bee samples, the mean number of spores across all sampled bees (NSS) was always much lower than the one calculated across infected bees only (NSI) (Table 3).

### Experiment 2

#### Nosema ceranae prevalence

**Fig 3:**
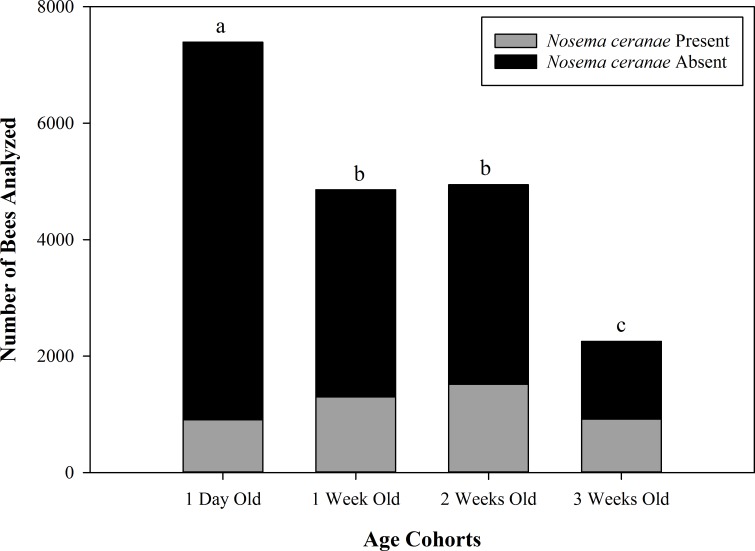
Prevalence of *Nosema ceranae* infection in bees of four age cohorts within experimental nucleus colonies. The proportion of bees infected with *Nosema ceranae* spores within different age cohorts are shown for all 8 quadruple-cohort colonies combined. Different letters indicate significant differences among the infection prevalences of age cohorts (P < 0.01). Age labels indicate age at colony establishment; bees were two weeks older when analyzed.

DNA analysis revealed that only *N*. *ceranae* was present in bee samples in all eight experimental nucleus colonies (S1 Fig). The total number of bees analyzed is summarized in Table 4 and the results of *Nosema ceranae* prevalence are summarized in Fig 3. Because the effect of age on *N*. *ceranae* prevalence was not dependent on the year (*F*_3, 18_ = 1.86; *P* = 0.1723), data from both years were combined for subsequent analyses. The prevalence of *Nosema ceranae* infection was significantly different between some age cohorts (*F*_3, 18_ = 75.64; *P* < 0.01). *Nosema ceranae* infection prevalence was significantly higher in 3 week old bees (38.78% infected) than in any other age cohort. One day old bees i.e. the age cohort that was actually 2 weeks old when analyzed had significantly lower prevalence (10% infected) of *Nosema ceranae* infection when compared to any other age cohort. There was no significant difference in infection prevalence between 1 week old bees (25.38% infected) and 2 week old bees (29.11% infected). Further, *N*. *ceranae* infection was not detected in any of the eight queens analyzed from the experimental nucleus colonies using spore analysis method.

**Table 4:** Summary of the number of bees analyzed in each of the eight quadruple-cohort colonies.

		Age of bees at colony establishment			
		1 Day	1 Week	2 Weeks	3 Weeks
Year	Hive	Number of Bees			
1	1	794	702	662	469
	2	854	452	600	248
	3	895	585	739	301
	4	1005	644	514	436
2	5	1051	691	823	298
	6	923	648	576	113
	7	826	625	550	258
	8	1044	512	480	130
	**Total:**	**7392**	**4859**	**4944**	**2253**

#### *Nosema ceranae* intensity

**Fig 4:**
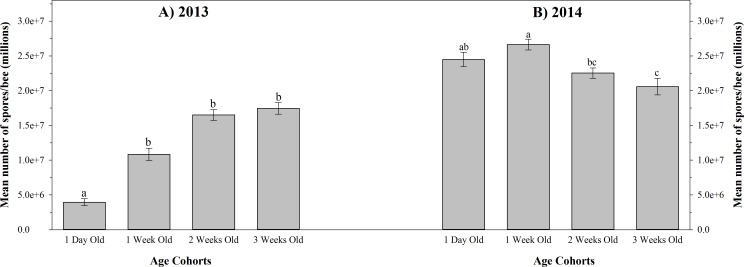
*Nosema ceranae* infection intensity in bees of four age cohorts within experimental nucleus colonies in 2013 and 2014. Mean ± SE *Nosema ceranae* infection intensities within different age cohorts are shown for (A) all four 2013 quadruple-cohort colonies combined, and (B) all four 2014 quadruple-cohort colonies combined. Different letters indicate significant differences between age cohorts (P < 0.01) of the same year. Age labels indicate age at colony establishment; bees were two weeks older when analyzed.

The results of *N*. *ceranae* intensity are summarized in Fig 4. The effect of age on *N*. *ceranae* intensity was dependent on the year (*F*_3, 18_ = 15.89; *P* < 0.01); therefore, data from each year were analyzed separately. Mean *Nosema ceranae* infection intensity was significantly different between some age cohorts in 2013 (*F*_3, 9_ = 14.72; *P* < 0.01). One day old bees had a significantly lower mean intensity of *N*. *ceranae* infection when compared to any other age cohort. The mean infection intensities in 3 week, 2 week, and 1 week old bees were not significantly different (Fig 4A). Mean *Nosema ceranae* infection intensities were also significantly different between some age cohorts in 2014 (*F*_3, 9_ = 5.38; *P* = 0.0213). Both 1 day old and 1 week old bees exhibited significantly higher mean spore intensities than the bees in 3 week old cohort. When compared to 2 week old bees, however, only the 1 week old cohort had a significantly higher spore intensity (Fig 4B).

#### Hypopharyngeal gland protein content

**Fig 5:**
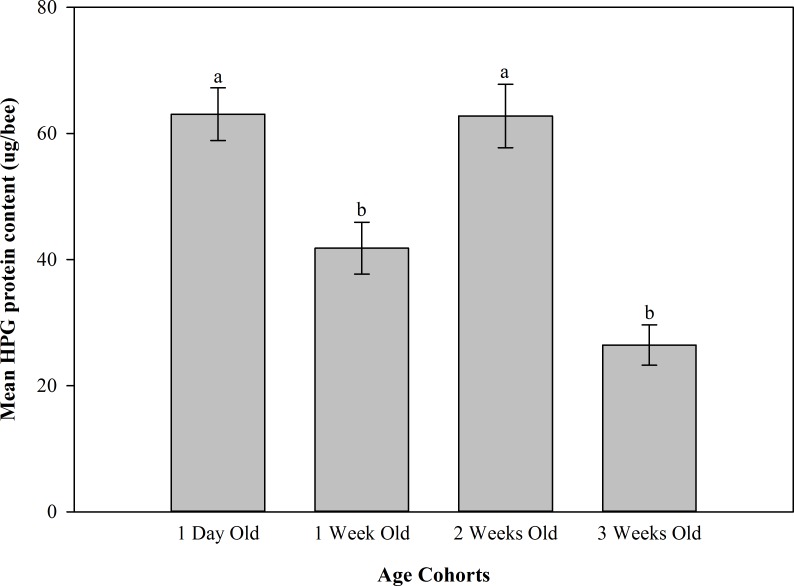
Hypopharyngeal gland protein content in bees of four age cohorts within experimental nucleus colonies in 2013. Mean ± SE hypopharyngeal gland protein content within different age cohorts are shown for all four of the 2013 quadruple-cohort colonies combined. Different letters indicate significant differences among the age cohorts (P < 0.0153). Age cohort labels indicate age at colony establishment. Bees of all cohorts were two weeks older when analyzed.

The results of hypopharyngeal gland protein content are summarized in Fig 5. The effect of age on hypopharyngeal gland protein content was not dependent on *N*. *ceranae* infection level (*F*_6, 17_ = 1.48; *P* = 0.2432); therefore, age and infection level data were combined for subsequent analyses. There were significant differences in mean hypopharyngeal gland protein concentration between some age cohorts (*F*_3, 9_ = 17.74; *P* = 0.0153). The hypopharyngeal glands of 1 day old bees (i.e. two weeks old when sampled) and 2 week old bees (i.e. 4 week old bees when sampled) contained significantly more protein (63.1±4.2 μg/bee (SE) and 62.8 ±5.0 μg/bee (SE), respectively) than those of 1 week old bees (i.e. 3 weeks old when sampled) (41.8 ±4.1 μg/bee (SE)) and 3 week old bees (i.e. 5 weeks old when sampled) (26.4 ±3.2 μg/bee (SE)). We found no significant differences in mean hypopharyngeal gland protein content among bees within the three levels of *N*. *ceranae* infection severity (*Nosema* free bees; low level of infection (7.5 x 10^5^–1.2 x 10^6^ spores) and high level of infection (3.685 x 10^7^–5.76 x 10^7^ spores); *F*_2, 6_ = 0.30; *P* = 0.7538). These infection levels were selected because, among the infected bees, most exhibited either a minor infection or severe infection, i.e. most infected bees had spore intensities that fell within the range of either the low infection level or the high infection level. The mean hypopharyngeal gland protein content (μg/bee) in bees with no *Nosema* infection, low level of infection and high level of infection was 44.4±3.7 (SE), 52.7±4.7 (SE) and 48.5±3.7 (SE) respectively.

#### Gut pH Analysis

As the effect of *N*. *ceranae* infection on mean gut pH did not depend on the year (*F*_1, 6_ = 0.29; *P* = 0.6126), data from both years were combined for subsequent analyses. There was no significant difference in mean gut pH of *N*. *ceranae* infected bees and non-infected bees (independent samples t-test, t = -0.31; df = 46; *P* = 0.756). The mean gut pH values pertaining to infected and non-infected bees were 6.09±0.11 (SE) and 6.04±0.11 (SE) respectively.

## Discussion

This study provides comprehensive insights into *N*. *ceranae* infection dynamics (prevalence and intensity) at the colony level, and also demonstrates that *N*. *ceranae* doesn’t have an effect on hypopharyngeal gland protein content and midgut pH. This is one of the few studies that have directly compared the efficacies of employing individual and composite bee samples (multiple bees per sample) for colony level diagnosis of *N*. *ceranae* infection [38, 40]. We found no correlation between the prevalence and intensity of *N*. *ceranae* infection in composite samples (Experiment 1). Further, our results suggest that the prevalence and intensity of *N*. *ceranae* infection is significantly influenced by honey bee age. These findings emphasize the risk of using composite samples (samples constituting multiple bees) to determine *Nosema* infection levels within a colony and the potential bias of collecting samples solely comprised of same aged bees. To our knowledge, this is the first study to analyze each individual bee from whole nucleus colonies infected by *N*. *ceranae* in order to gain a comprehensive understanding of the colony level infection dynamics (Experiment 2).

In the first experiment, there was no significant correlation between *N*. *ceranae* infection intensity and percentage of infected bees in composite samples. These results further indicate the risk of using spore counts from composite samples (samples containing multiple bees) for making inferences regarding *N*. *ceranae* infection in colonies. It may not be possible to accurately estimate *N*. *ceranae* infection prevalence based on infection intensity in composite samples. Hence, it appears that analysis of individual bee samples would be ideal to accurately diagnose a colony’s infection level even though it is more time-consuming and expensive, especially if using the PCR method for analysis.

Furthermore, the *N*. *ceranae* infection prevalence values from composite samples of background bees were not significantly different than those of the marked-bee age cohorts specific to each sampling date, and appear to lie between the prevalence values of nurse-aged and foraging-aged bees (Fig 1). These results support the assertion made by Smart and Sheppard [38] and Traver et al. [13] that a bee sample containing a mixture of ages provides a better representation of colony’s infection level. Although the results from both Experiment 1 and 2 indicate a lower *N*. *ceranae* infection prevalence in nurse-aged bees than in foraging-aged bees, the infection is still likely to be detected by sampling nurse bees; therefore, nurse bees should be represented in colony infection diagnosis. Forager bees only represent a portion of the colony; but in this experiment, the odds of infection in foraging-aged bees were significantly higher than in other age cohorts. Therefore, exclusively sampling forager bees may bias the sample to the most severely infected bees and may create an inaccurate impression of colony infection level. On the other hand, sampling only nurse bees may create a perceived infection level that is lower than what the colony as a whole is experiencing. Other factors such as climate [56], season [13, 43], exposure to chemicals [31, 57, 58] and time of sampling [44] may also influence the proportion of infected bees in a sample and should also be considered for establishing a robust sampling protocol.

In Experiment 2, there were major differences in *N*. *ceranae* intensity between the experimental colonies established in 2013 and 2014 (Fig 4). This discrepancy can likely be explained by the time of year in which these colonies were established and sampled, in turn influencing the diets to which these colonies would have been exposed. Several studies have demonstrated that bees with a greater quantity of pollen in their diet have increased *N*. *ceranae* spore intensities [32, 59–61]. The 2014 experimental colonies were established in May whereas the 2013 colonies were established in late July when fewer plants species offering pollen were in bloom. The lower amount of pollen available to the experimental colonies in 2013 likely contributed to lower spore intensities.

Additionally, bees from the 1 day old and 1 week old cohorts of 2014 demonstrated the highest *N*. *ceranae* intensities (Fig 4B). This again may be a result of differences in diet among the age cohorts. Crailsheim et al. [62] reported that nurse bees at 4 and 9 days old had the highest amount of pollen in their gastrointestinal tracts and foragers only contained minimal amounts of pollen. Thus, when we inoculated our experimental colonies with *N*. *ceranae*, the 1 day and 1 week old cohorts (3 days and 10 days old at the time of inoculation, respectively) may have more recently consumed pollen when compared to foragers; potentially further exacerbating *N*. *ceranae* spore intensities. Bees in older cohorts likely would have greatly reduced their pollen consumption, as well as taken cleansing flights—thereby reducing the amount of spores present in their gut at the time of sampling. Similar results were not observed in the 2013 experimental colonies (Fig 4A) as these colonies were both sampled and established at a different time of the year (July), when pollen forage was relatively scarce.

Further, the studies that found *N*. *ceranae* intensity to increase as a result of high pollen in the diet also found that the bees fed with high pollen diets survived better than the bees that received low pollen diets, despite higher spore intensities [32, 59–61]. In an earlier study we demonstrated that *N*. *ceranae* prevalence is not influenced by diet [32], which suggests that prevalence may be a more reliable indicator of colony infection status, as infection prevalence does not appear to fluctuate based on the nutritional status of the bees. Therefore, we propose that measuring the prevalence of infection in individual bees of mixed ages may provide the most accurate diagnosis of a colony’s *N*. *ceranae* infection levels. Further research using this proposed colony diagnostic method is critical to ascertain the necessary amount of individual bees to be analyzed for an accurate diagnosis of the disease and to determine the infection thresholds at which to begin treatment for *N*. *ceranae* infection.

In our study (both experiments), we found that foraging-aged bees had a higher prevalence of *N*. *ceranae* infection when compared to nurse-aged bees (Figs 1 and 3). These results are in agreement with findings of several other studies [2, 38, 44–47]. This phenomenon may be a result of differential spore production in nurse- and forager-aged bees. In some nurse-aged bees, spores may not have been produced yet, as compared to forager-aged bees in which spores may have had ample time to develop. Further, Schmid-Hempel [63] explains that diseases are more likely to be picked up by foragers, while young workers are at less risk of infection.

It is presumed that the oral transfer of food is the primary mode of *N*. *ceranae* infection transmission between bees [64]; in this manner, queens become infected from sick attending bees [65]. However, none of the queens in our study (Experiment 2) tested positive for *N*. *ceranae* infection. This is likely due to the low prevalence of infected bees in the 1 day old cohort (which were two weeks old at the time of sampling), as these would have been the nurse bees feeding the queen [66, 67]. In contrast to our results, Higes et al. [65] found the queens of their cage experiment were heavily infected 21 days after exposure to *N*. *ceranae* infected workers. Three factors may explain this disparity: a shorter length of our study (our colonies were euthanized 14 days after inoculation), a lower infection intensity in the queen attendants in our study (in year 2013) compared to Higes et al. [65] (infection prevalence was not measured by Higes et al. [65]), and the experimental design (our experimental colonies were in the field, while Higes et al. [65] placed newly emerged workers and queens directly into cages). Determining the minimum infection prevalence in workers attending the queen that leads to queen infection should be a targeted objective of future research, as it would greatly benefit beekeepers in their colony management decisions. Further, it should be noted that spore counts may underestimate the actual number of bees infected with *N*. *ceranae* as some bees may not have spores yet but have vegetative stages.

Our results suggest that *N*. *ceranae* did not have a significant effect on hypopharyngeal gland protein content. Previous studies with caged bees have demonstrated negative effects of *N*. *ceranae* on hypopharyngeal glands; one study reported a reduction in gland size [31] and another showed a significant reduction in protein content [32]. Hypopharyngeal gland development is influenced by the age/role of workers [68], their diet [69], and the presence of brood [70, 71]. Hence, hypopharyngeal gland protein content is influenced by several factors. This may explain the discrepancy between our findings and the results from above mentioned studies, as the rearing environments were different in these studies. In our study the bees were in colonies under natural field conditions, whereas bees were reared in a cage environment in others. Further, as reduction in hypopharyngeal gland protein from *N*. *ceranae* infection was observed in laboratory conditions but not in field conditions, we may speculate that stimuli affecting protein synthesis in hypopharyngeal glands might exist in field colonies and may offset the negative impact of *N*. *ceranae* infection on hypopharyngeal gland protein. In this study it was not possible to directly compare the hypopharyngeal gland protein between uninfected and infected bees. This fact should also be kept in mind when interpreting the hypopharyngeal gland protein data. However, further research is needed to understand how above mentioned factors mitigate the effects of *N*. *ceranae* on the hypopharyngeal glands of bees in the field.

Nurse bees have well-developed and highly active hypopharyngeal glands [70, 72] that produce and secrete most of the proteins contained in royal jelly [73,74]. This was, in fact, corroborated in our study as the 1 day old cohort (2 weeks old at time of analysis) had the highest mean concentration of hypopharyngeal gland protein (Fig 5). Surprisingly, bees in the 2 week old cohort exhibited high protein concentrations in the hypopharyngeal glands similar to the bees in the 1 day old cohort. These bees were 4 weeks old at the time of analysis and were expected to have transitioned to foraging tasks. The only plausible explanation for this unexpected finding is that the foraging bees may have reverted back to nursing tasks to adjust the brood rearing work force, thereby meeting the needs of increasing brood in the colony. Sagili et al. [52] have shown that high brood pheromone in a colony signals the presence of greater number of larvae, and the bees in a high brood pheromone environment have higher hypopharyngeal gland protein.

## Supporting Information

S1 Fig Gel showing multiplex PCR amplicons of lengths specific to the 16S rRNA of *Nosema ceranae* and *Nosema apis*, as well as the honey bee house-keeping gene RpS5. Lanes 1–8 show samples from infected honey bees from each of the eight quadruple-cohort colonies. Only the *Nosema ceranae* species was present in the colonies. Lane 9 is a sample infected only with *Nosema ceranae*, Lane 10 is a sample infected only with *Nosema apis*. Lane 11 is a sample of non-infected honey bees. Lane M is a 100 bp DNA ladder.
(TIFF)
