# Supplementary figures and images for: Colony Level Prevalence and Intensity of Nosema ceranae in Honey Bees (Apis mellifera L.)
Source: PLoS One. 2016 Sep 22;11(9):e0163522. doi: 10.1371/journal.pone.0163522 (PMC5033419; doi:10.1371/journal.pone.0163522)

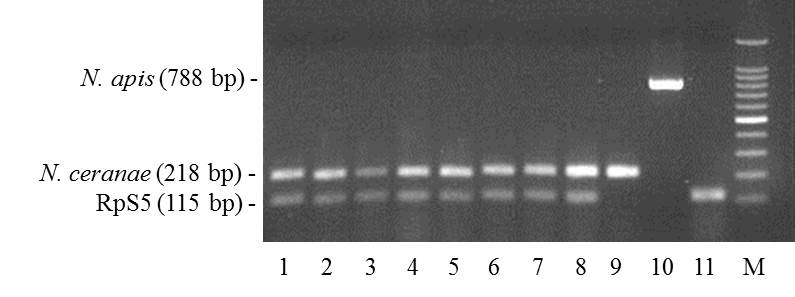

Supplement: S1 Fig — Lanes 1–8 show samples from infected honey bees from each of the eight quadruple-cohort colonies. Only the Nosema ceranae species was present in the colonies. Lane 9 is a sample infected only with Nosema ceranae, Lane 10 is a sample infected only with Nosema apis. Lane 11 is a sample of non-infected honey bees. Lane M is a 100 bp DNA ladder. (TIFF) [file pone.0163522.s001.tiff]
